# Current knowledge of Chagas-related heart disease among pediatric cardiologists in the United States

**DOI:** 10.1186/s12872-021-01924-8

**Published:** 2021-03-02

**Authors:** Sanchi Malhotra, Imran Masood, Noberto Giglio, Jay D. Pruetz, Pia S. Pannaraj

**Affiliations:** 1 Division of Infectious Diseases at Children’s Hospital Los Angeles, 4650 Sunset Blvd MS #51, Los Angeles, CA 90027 USA; 2 Division of Cardiology at Children’s Hospital Los Angeles, Los Angeles, CA USA; 3 Epidemiología Hospital de Niños Ricardo Gutierrez, Buenos Aires, Argentina; 4 Keck School of Medicine, University of Southern California, Los Angeles, CA USA

**Keywords:** Chagas heart failure, Pediatrics, Cardiomyopathy, Conduction, Epidemiology

## Abstract

**Background:**

Chagas disease is a pathogenic parasitic infection with approximately 8 million cases worldwide and greater than 300,000 cases in the United States (U.S.). Chagas disease can lead to chronic cardiomyopathy and cardiac complications, with variable cardiac presentations in pediatrics making it difficult to recognize. The purpose of our study is to better understand current knowledge and experience with Chagas related heart disease among pediatric cardiologists in the U.S.

**Methods:**

We prospectively disseminated a 19-question survey to pediatric cardiologists via 3 pediatric cardiology listservs. The survey included questions about demographics, Chagas disease presentation and experience.

**Results:**

Of 139 responses, 119 cardiologists treat pediatric patients in the U.S. and were included. Most providers (87%) had not seen a case of Chagas disease in their practice; however, 72% also had never tested for it. The majority of knowledge-based questions about Chagas disease cardiac presentations were answered incorrectly, and 85% of providers expressed discomfort with recognizing cardiac presentations in children. Most respondents selected that they would not include Chagas disease on their differential diagnosis for presentations such as conduction anomalies, myocarditis and/or apical aneurysms, but would be more likely to include it if found in a Latin American immigrant. Of respondents, 87% agreed that they would be likely to attend a Chagas disease-related lecture.

**Conclusions:**

Pediatric cardiologists in the U.S. have seen very few cases of Chagas disease, albeit most have not sent testing or included it in their differential diagnosis. Most individuals agreed that education on Chagas disease would be worth-while.

## Background

Chagas disease is a pathogenic parasitic infection caused by *Trypanosoma cruzi,* with a prevalence of approximately 8 million cases worldwide and greater than 300,000 cases in the United States of America (U.S.) [1]. Chagas disease is endemic to Latin American and well known to cause significant cardiac complications. About 50% of immigrants to the U.S. are from highly endemic Latin American countries including Mexico and may carry the parasite with them occultly [2, 3]. In the U.S., Chagas disease is primarily acquired vertically during childbirth causing congenital Chagas disease [4–6]. An estimated 40,000 women of childbearing age in the U.S. are infected with Chagas disease, with a 1–5% risk of vertical transmission, and up to 300 congenital cases in the U.S. annually [2, 7, 8]. Chagas disease can be transmitted through blood transfusion or organ donation, though this risk is decreased with implementation of screening programs [9]. In most rural parts of Latin America, Chagas disease is mainly transmitted through the feces of the vector, the triatomine bug, which is also present in the southern half of the U.S. Despite the prevalence of Chagas disease in the United States, it is rarely considered for patients with otherwise unexplained cardiac disease [2, 3].

The impact of this disease can be extensive and difficult to treat with 30% of infected persons developing long term consequences, most importantly cardiomyopathy [1, 4]. Although congenital Chagas disease and/or pediatric Chagas disease cases comprise a small proportion of cases, a survey of U.S. obstetricians and pediatric infectious disease doctors showed a lack of comfort and knowledge of the disease presentations [7, 10]. Pediatric cardiologists may be the first point of contact in children with undiagnosed Chagas disease; therefore, knowledge of its clinical presentation and epidemiology is essential for these providers. The earlier the presence of the parasite is recognized and treated, the better the outcomes; therefore, pediatric recognition and diagnosis of Chagas disease would reduce morbidity and mortality [11].

The cardiac presentations of Chagas disease can be highly variable in pediatrics and limited data are available regarding the cardiac status of children in the U.S. with Chagas disease. Data from endemic countries shows cardiac presentations in neonates and infants with congenital Chagas disease, to adolescents showing pathologic and non-pathologic electrocardiographic changes, and even some adolescents with signs of cardiomyopathy [12–20]. Congenital Chagas disease is typically asymptomatic, but can present with heart failure, electrocardiogram (EKG) changes or myocarditis. When congenital Chagas is fatal, it is most often related to myocarditis or meningoencephalitis [8, 20]. Two infants with congenital Chagas disease recognized in the U.S. presented with hydrops fetalis [8]. Acute Chagas disease can present with myocarditis in any age group as well [14]. A metanalysis found that EKG changes in children and adolescents are found just as frequently as in adults but with more rapid time to death and more diagnostic difficulty [15]. The most common findings are right bundle branch block, atrioventricular block and left anterior fascicular block [15, 19, 21]. A study in Mexico looked at 37 cases of Chagas disease in adolescents under 18 and found that 25 of them already had cardiac pathology consistent with Chagas disease cardiomyopathy [12].

The purpose of our study is to survey pediatric cardiologists to assess their knowledge, awareness and practice with the recognition and management of Chagas disease. Our hope is to identify gaps in knowledge and comfort level of pediatric cardiologists, so that specific educational curriculum can be designed to increase diagnostic consideration of Chagas disease and initiate earlier treatment.

## Methods

We performed a prospective, quantitative, descriptive study. Our target survey population was cardiologists who care for pediatric patients in the U.S. A questionnaire including demographic, multiple choice and likert-scale questions was administered to the survey population via the PediHeart, Western Society of Pediatric Cardiology and Pediatric CHF listservs. There may be an overlap in recipients on the listservs. The PediHeart listserv, which was our most inclusive group, contained 1912 members of which 1205 are physicians practicing in the U.S. which was our target audience. These listservs are open to medical professionals, who voluntarily subscribe. Our questions were developed with the input of pediatric cardiology and pediatric infectious disease specialists, and three experts in Chagas disease (from the U.S. and Argentina).

The survey was emailed via these listservs via Qualtrics (Provo, UT) using an anonymous link (Additional file 1: Figure S1). The survey was sent out once a month for 3 months from August 2019 to November 2019 (prior to the Coronavirus Disease 2019 pandemic), with one initial request, and two reminder emails. The recipients were offered the chance to enter a raffle for three $100 gift cards if they chose to give their email address which was not linked to their survey response. We requested that the survey be completed without any external aids within the 3-month period. The study was approved by the Children’s Hospital Los Angeles institutional review board. Informed consent was not required, but a research information sheet was sent with the study (Additional file 2: Figure S2).

All data was aggregated by the authors of this paper. We excluded cardiologists who do not see any pediatric patients or who do not practice in the U.S. Statistical analysis provided by Qualtrics was used. Additional statistical evaluation was performed using JMP (Version 15). To compare responses, chi-square analysis was performed for categorical variables and t-test was performed for continuous variables as appropriate. All authors had full access to all the data in the study and take responsibility for its integrity and the data analysis.

## Results

**Fig. 1:**
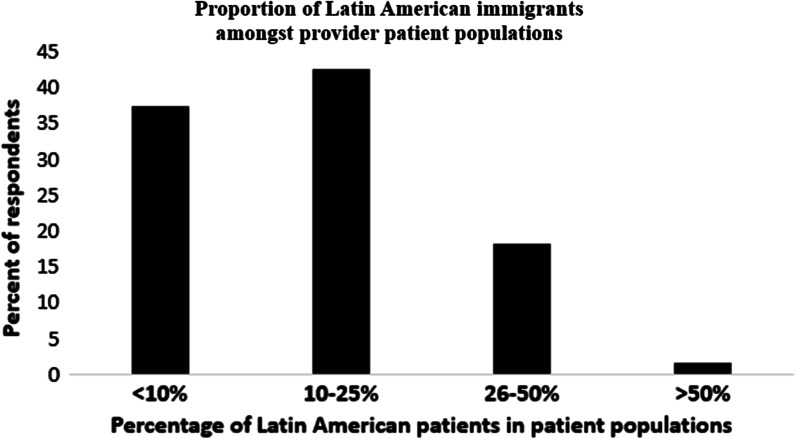
Latin American patient population of survey respondents. We found that 62.5% of our respondents have a > 10% Latin American patient population who may be at risk for Chagas

**Fig. 2:**
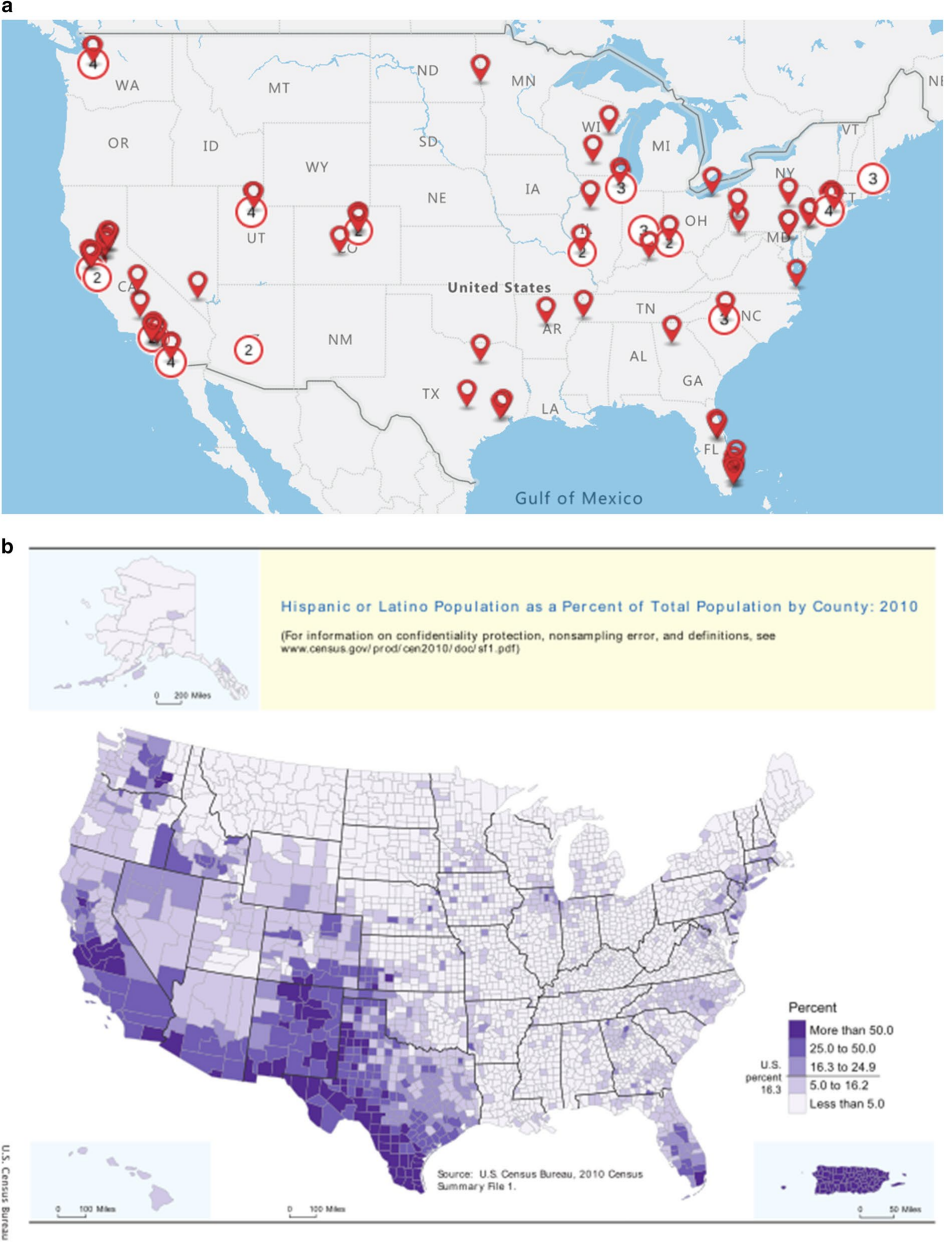
**a** Map of study survey respondents by zipcode, with many respondents in areas with significant Latin American populations (compared to 2B) and in areas where triatomine bug is present (see triatomine bug map at https://www.cdc.gov/parasites/chagas/gen_info/vectors/index.html/ [1]). **a** was made by the authors. **b** Map of US Latin American population by zip code. **b** was adapted from the US Census Bureau

We received responses from 139 cardiologists. Twenty cardiologists practiced outside of the US and were excluded. The remaining 119 surveys from cardiologists who treated pediatric patients in the US were included in the analysis. Of these, 17 practitioners cared for both adult and pediatric patients. The majority of respondents were general cardiology practitioners located in an academic setting (Table 1) and 62.5% of respondents served a > 10% Latin American patient population determined by their zip codes (Figs. 1, 2). Poor knowledge of vector presence in the U.S. and potential future risk of Chagas disease transmission was noted in our study. While the vector was found in 82% of our respondents’ states, only 20% (95% CI 13.5–27.8) reported that the vector was present [1].

**Table 1:** Demographics of 119 pediatric cardiologist survey respondents

**Pediatric cardiac specialty**		**Practice setting**	
General cardiology	91 (75.8%)	Academic	79 (65.8%)
Imaging	48 (40%)	Public hospital	40 (33.3%)
Fetal medicine	36 (30%)	Private hospital	29 (24.2%)
Heart failure	18 (15%)	Group practice	30 (25%)
Heart transplant	16 (13.3%)	Individual practice	1 (0.8%)
Cardiothoracic ICU	15 (12.5%)	Retired	1 (0.8%)
Adult congenital heart disease	15 (12.5%)	Other	1 (0.8%)
Cardiac catheterization	11 (9.2%)		
Electrophysiology	6 (5%)	**Years in practice**	
Other	2 (1.7%)	0–5	56 (46.7%)
		6–10	22 (18.3%)
		11–15	8 (6.7%)
		15+	34 (28.3%)

Experience with Chagas disease appeared to be minimal, and testing was rarely performed. Of all respondents, 87% (95% CI 80.1–92.7) had not seen a case of Chagas disease in their practice; however, 72% (95% CI 62.8–79.6) had never tested for it. In our sample, 27% (95% CI 19.6–36.2) of respondents had tested for Chagas disease between 1 and 10 times in their career and only one respondent had tested for Chagas disease greater than 10 times. Cases of Chagas disease seen did not differ based on years in practice, with newer physicians and those in practice for more than 10 years having similar rates (*p* = 0.98). Multiple choice questions were utilized to ascertain knowledge of basic cardiac presentations of Chagas (Table 2) [7, 21]. Only 1 of 4 knowledge-based questions was answered correctly by greater than 50% of respondents, while the rest had a less than 30% correct response rate. Correct responses to the four multiple choice questions also did not differ by respondents’ years in practice (*p* = 0.104, *p* = 0.67, *p* = 0.92, *p* = 0.51).

**Table 2:** Survey questions about baseline Chagas disease knowledge

Survey question	Correct answer	% Respondents answered correctly
What is the most common echo finding in Chagas cardiomyopathy?	Apical aneurysm	52% (95% CI 42–60.8)
What is the most common EKG finding in Chagas cardiomyopathy?	Right bundle branch block	25% (95% CI 17.5–33.8)
Which test should be sent as the initial screen for Chagas?	T. cruzi ELISA assay	26% (95% CI 18.5–35.1)
What is the most common cause or death from Chagas disease?	Tachyarrhythmia	22% (95% CI 14.9–30.5)

When asked about congenital Chagas disease, only 57.5% (95% CI 48.6–66) of respondents said they would test for Chagas disease in a newborn with a maternal history of positive *T. cruzi* serology. However, 52.9% (95% CI 43.6–61.2) of respondents did recognize a preterm neonate with hepatosplenomegaly, myocarditis and cardiac insufficiency as a possible presentation of congenital Chagas disease. While 83.3% (95% CI 75.7–88.9) and 75.6% (66.6–81.9) of respondents recognized myocarditis and conduction anomalies as presentations of acute Chagas infection respectively, only 41.7% (95% CI 33.2–50.6) of respondents recognized mural thrombosis as a possible acute presentation. Only 7% (95% CI 3–13.2) of respondents reported being familiar with the 2018 American Heart Association (AHA) published scientific statement regarding Chagas disease. Familiarity with the AHA statement did not differ based on respondents’ years in practice (*p* = 0.51) [21].

**Fig. 3:**
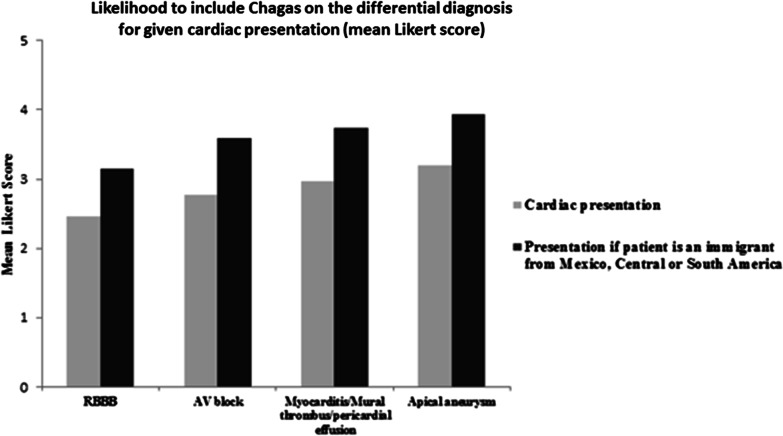
Percentage of respondents who have ever tested for Chagas disease and likelihood of including Chagas disease in the differential diagnosis for associated cardiac conditions. Respondents were asked how likely they were to include Chagas disease on the differential for a given cardiac finding. A follow-up question for each presentation then asked how likely Chagas disease would be on the differential for a patient who recently immigrated from Mexico, Central or South America. Likert scale was coded from 1 to 5 (1 = strongly disagree, 2 = somewhat disagree, 3 = neither agree nor disagree, 4 = somewhat agree, 5 = strongly agree). Respondents were more likely to include Chagas disease on the differential if the patient was an immigrant from Mexico, Central or South America for each given symptom

From the Likert scale questions, 85% of respondents disagreed with the statement: “I feel comfortable recognizing cardiac presentations of Chagas disease in children”. Most respondents selected that they would not include Chagas disease on their differential diagnosis for various cardiac presentations that included conduction anomalies, mural thrombus, myocarditis and/or apical aneurysms (Fig. 3). However, when considering patients who recently immigrated from Latin American nations, inclusion of Chagas disease in the differential diagnosis increased. In response to the statement: “If I was offered a lecture on Chagas-related heart disease, I would be likely to attend,” 87% of respondents agreed. In addition, 64% of respondents stated that education on Chagas disease would lead to practical knowledge for their clinical practice which is consistent with 62.5% of respondents living in an area with > 10% Latin American population.

## Discussion

To our knowledge, this is the first study performed to assess knowledge and comfort amongst pediatric cardiologists regarding Chagas related heart disease. Overall, our results demonstrate that pediatric cardiologists are unfamiliar and uncomfortable with diagnosing and managing Chagas related heart disease, while recognizing their need to learn more.

A study in Los Angeles community centers found prevalence of *T. cruzi* to be as high as 4.7% of immigrants from specific Latin American countries [22]. For children who are immigrating from Chagas-endemic Latin American countries, Chagas disease should be a serious consideration for neonates and adolescents with compatible cardiac presentations. Expecting mothers from endemic countries should also be recognized as high risk to vertically transmit Chagas disease to their newborns. Given that the U.S. lacks routine perinatal screening for Chagas compared to Chagas-endemic Latin American countries, U.S. providers must be at high alert as early treatment could prevent development of cardiac sequelae. Our study found that Chagas disease was more frequently considered in immigrants, but it was still unlikely to be in the providers’ differential diagnosis overall. Also, 72% of respondents had never sent diagnostic testing for Chagas disease despite a majority of our respondents practicing in locations with high number of Latin American immigrants. Familiarity with Chagas disease did not change with years of practice, which may indicate that despite increases in immigration over the past few decades, consideration of Chagas disease for our patients has not changed.

The AHA published a statement paper regarding Chagas disease in 2018, with which only 7% of our respondents were familiar [21]. This unfamiliarity is likely multifactorial. Educational factors as well as low experience with this disease likely play a role. Traditional teaching about Chagas disease informs providers that the *T. cruzi* parasite is both endemic and limited to Latin America. As immigration from Latin American countries increase, improved provider awareness is needed. Lack of awareness in our respondents likely results in less frequent testing for Chagas disease, and may ultimately lead to an underestimation of the true prevalence of Chagas in the U.S. pediatric population. We did find that most of our respondents were interested in learning more, with many respondents asking for the answer key after completing the survey. Future steps for our study would be to create and disseminate educational material especially in areas of higher prevalence to help ensure quicker diagnosis and referral to appropriate care for patients.

The most important limitation of this study is our low response rate of about 10%. This is likely due to unfamiliarity or lack of interest in the topic and possibly the length of the survey. However, we believe that the cardiologists who answered the survey were likely to have more interest in Chagas disease. Another limitation in our study was using a raffle as an incentive to fill out the survey as it may have financially motivated respondents. Overall, our results likely underestimate the true lack of knowledge on this topic and there may be an even greater need for education.

## Conclusions

Pediatric cardiologists in the U.S. have seen very few cases of Chagas disease, but very few have tested for it or included it in their differential diagnosis. However, most cardiologists agreed that learning more about Chagas disease would be worth-while. Future studies involving a medical curriculum should focus on increasing recognition, testing and early treatment of Chagas in the U.S.

## Supplementary Information

**Additional file 1: FigureS1:** Complete survey sent to participants with correct answers.

**Additional file 2: FigureS2:** Research Information Sheet.

## Data Availability

The datasets used and/or analyzed during the current study available from the corresponding author on reasonable request.

## Abbreviations

U.S.: United States
EKG: Electrocardiogram
AHA: American Heart Association
