# Supplementary material for: Current knowledge of Chagas-related heart disease among pediatric cardiologists in the United States
Source: BMC Cardiovasc Disord. 2021 Mar 2;21:116. doi: 10.1186/s12872-021-01924-8 (PMC7921824; doi:10.1186/s12872-021-01924-8)
Supplement: Supplementary file 1 — Additional file 1: FigureS1: Complete survey sent to participants with correct answers. [file 12872_2021_1924_MOESM1_ESM.docx]

Journal: Pediatric Cardiology

Current knowledge of Chagas-related heart disease among pediatric cardiologists in the United States

Sanchi Malhotra MD^1^, Imran Masood DO^2^, Noberto Giglio MD^3^, Jay D. Pruetz MD^2,4^, Pia S. Pannaraj MD, MPH^1,4^

^1^Division of Infectious Diseases and ^2^Cardiology at Children’s Hospital Los Angeles, California, USA; ^3^Epidemiología Hospital de Niños Ricardo Gutierrez, Buenos Aires, Argentina; ^4^Keck School of Medicine, University of Southern California, Los Angeles, California, USA

Corresponding Author:

Sanchi Malhotra MD

Email: [samalhotra@chla.usc.edu](about:blank)

Supplemental Fig 1. Complete survey sent to participants with correct answers

Start of Block: Default Question Block

| 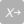 |
| --- |

Q1 In which country do you currently reside?

▼ United States of America (1) ... Zimbabwe (1357)

Q2 Please enter your zip code

________________________________________________________________

Q3 As a cardiologist, I primarily treat:

- Adult patients (1)
- Pediatric patients (2)
- Both adult and pediatric patients (3)

Q4 Please select the following specialty that applies to you (select all that apply):

- General Cardiology (1)
- Imaging (2)
- Cardiac Catheterization (3)
- Fetal Medicine (4)
- Adult Congenital Heart Disease (5)
- Electrophysiology (6)
- Heart Failure (7)
- Heart Transplant (8)
- Cardiothoracic ICU (9)
- Other (10)

Q5 Please select the setting in which you practice (select all that apply):

- Hospital (private) (1)
- Hospital (public) (2)
- Group practice (3)
- Individual practice (4)
- Academic (5)
- Retired (6)
- Other (7)

Q6 How many years have you been in practice?

- 0-5 years (1)
- 6-10 years (2)
- 10 -15 years (3)
- 15 or more years (4)

Q7 What percentage of your patients have immigrated to the United States from a Latin American country (including Mexico, Central America and South America)?

- (1)
- 10%-25% (2)
- 26%-50% (3)
- >50% (4)

Q8 The vector for Chagas disease (kissing bug) is found in my state

- Yes (1)
- No (2)

Q9 How many cases of Chagas disease have you seen in your practice?

- 0 (1)
- 1-5 (2)
- 6-10 (3)
- >10 (4)

Q10
How often have you tested for Chagas infection when working up a patient with cardiac symptoms?

- Never (1)
- 1-10 times (2)
- 10-100 times (3)
- >100 times (4)

Q11 Which of the following can be cardiac presentations of acute Chagas infection?

- **Myocarditis (1)**
- Coronary aneurysm (2)
- **Conduction anomalies or arrhythmias (3)**
- **Mural thrombosis (4)**
- Myocardial infarction (5)

Q12 What is the most common cause of death in Chagas cardiomyopathy?

- **Tachyarrhythmia (1)**
- Heart failure (2)
- Thromboembolic event (3)
- Cardiac tamponade (4)

Q13 Which of the following echo findings is most pathognomonic of Chagas cardiomyopathy?

- Intracardiac mural thrombus (1)
- Right atrial enlargement (2)
- **Apical aneurysm (3)**
- Septal enlargement (4)

Q14 Which of the following EKG findings is most suggestive of Chagas disease?

- Sinus node dysfunction (1)
- 3rd degree AV block (2)
- Atrial fibrillation (3)
- **Complete RBBB (4)**

Q15 I would send the following test as a screen for Chagas disease.

- Blood smear (1)
- T. Cruzi PCR (2)
- **T. Cruzi ELISA assay (3)**

Q16 What is the most common form of transmission of Chagas disease in cities?

- blood transfusion (1)
- **maternal fetal transmission (2)**
- contact with vector fecal material (3)
- organ transplantation (4)

Q17
What symptoms would make you think of testing for Chagas disease? (can select multiple)

- **a newborn with maternal history of positive Chagas serology (1)**
- a term infant with anemia and cardiac tamponade (2)
- **a pre-term infant with hepatosplenomegaly, myocarditis and cardiac insufficiency (3)**
- a teenager with prolonged QT (4)

Q18 I am familiar with the AHA statement for Chagas disease in the United States.

- Yes (1)
- No (2)

End of Block: Default Question Block

Start of Block: Block 1

| Q19 | Strongly agree (1) | Somewhat agree (2) | Neither agree nor disagree (3) | Somewhat disagree (4) | Strongly disagree (5) |
| --- | --- | --- | --- | --- | --- |
| I feel comfortable recognizing cardiac presentations of Chagas’ disease in children. (1) |  |  |  |  |  |
| If I encounter a patient found to have an apical aneurysm, I am likely to include Chagas disease on my differential. (2) |  |  |  |  |  |
| If I encounter a patient who immigrated from Mexico, Central or South America found to have an apical aneurysm, I am likely to include Chagas disease on my differential. (3) |  |  |  |  |  |
| If I encounter a patient found to have complete right bundle branch block, I am likely to include Chagas disease on my differential. (4) |  |  |  |  |  |
| If I encounter a patient who has immigrated from Mexico, Central or South America found to have right bundle branch block, I am likely to include Chagas disease on my differential. (5) |  |  |  |  |  |
| If I encounter a patient with new onset AV block, I am likely to include Chagas disease on my differential. (6) |  |  |  |  |  |
| If I encounter a patient who has immigrated from Mexico, Central or South America with new onset AV block, I am likely to include Chagas disease on my differential. (7) |  |  |  |  |  |
| If I encounter a patient with acute myocarditis, mural thrombus or pericardial effusions, I am likely to include Chagas on my differential. (11) |  |  |  |  |  |
| If I encounter a patient with acute myocarditis, mural thrombus or pericardial effusions who has recently immigrated from Mexico, Central or South America, I am likely to include Chagas on my differential. (8) |  |  |  |  |  |
| If I was offered a lecture on Chagas-related heart disease, I would be likely to attend. (9) |  |  |  |  |  |
| Education on Chagas-related heart disease would be practical knowledge for my practice. (10) |  |  |  |  |  |

End of Block: Block 1

Start of Block: Block 2

Q20 Please enter your email address if you would like to be entered in a raffle to win a $100 gift card. This is entirely optional. Your survey response will not be linked.

________________________________________________________________

End of Block: Block 2
