# Supplementary material for: Current knowledge of Chagas-related heart disease among pediatric cardiologists in the United States
Source: BMC Cardiovasc Disord. 2021 Mar 2;21:116. doi: 10.1186/s12872-021-01924-8 (PMC7921824; doi:10.1186/s12872-021-01924-8)
Supplement: Supplementary file 2 — Additional file 2: FigureS2: Research Information Sheet. [file 12872_2021_1924_MOESM2_ESM.docx]

Journal: Pediatric Cardiology

Current knowledge of Chagas-related heart disease among pediatric cardiologists in the United States

Sanchi Malhotra MD^1^, Imran Masood DO^2^, Noberto Giglio MD^3^, Jay D. Pruetz MD^2,4^, Pia S. Pannaraj MD, MPH^1,4^

^1^Division of Infectious Diseases and ^2^Cardiology at Children’s Hospital Los Angeles, California, USA; ^3^Epidemiología Hospital de Niños Ricardo Gutierrez, Buenos Aires, Argentina; ^4^Keck School of Medicine, University of Southern California, Los Angeles, California, USA

Corresponding Author:

Sanchi Malhotra MD

Email: [samalhotra@chla.usc.edu](about:blank)

Supplemental Figure 2. Research Information Sheet

Children’s Hospital Los Angeles

**RESEARCH INFORMATION SHEET**

Chagas Disease Survey Among Pediatric Cardiologists in the US vs Argentina

You are invited to participate in a research study conducted by Sanchi Malhotra, MD and colleagues from the Pediatric Residency Program and Cardiology Department at Children’s Hospital Los Angeles (CHLA) and in collaboration with Dr. Norberto Giglio from the Hospital del Niño’s Ricardo Gutierrez in Argentina. Participation in this study is completely voluntary.

The purpose of the study is to assess the knowledge, comfort, and experience of pediatric cardiologists with Chagas disease and the cardiac presentations. If you volunteer to participate in this study, your participation will involve completing an anonymous on-line survey. You will have 3 months to complete the survey and your participation in this study ends once you submit your responses.

This research involves the potential risk of accidental release of confidential information. You will be asked to provide your zip code (if in the US) or city (if in Buenos Aires) of where you practice cardiology; however, this information will not be linked to your name, email, or other identifying information that could directly identify you. You should not expect any direct benefit as a result of participating in this research. However, we hope that the information learned may increase knowledge and awareness about how comfortable pediatric cardiologists are with Chagas cardiomyopathy. The alternative to participation is to not participate.

There are no cost to you for participation in this study nor will you be paid for participation.

Only the research team will have access to the information you provide. None of the information will be disclosed to others; except if necessary to protect your rights or welfare or if required by law (i.e., harm to self or others, reports of certain infectious diseases). You will not be identified in publications of the research results. Authorized representatives of the CHLA Institutional Review Board may review subject records but are bound by rules of confidentiality.

Your choice about whether or not to participate will have no effect on your relationship with Children’s Hospital Los Angeles or Hospital del Niño’s Ricardo Gutierrez. If you agree to participate, but later decide to withdraw from this study, you may do so without affecting your relationship with CHLA or Hospital del Niño’s Ricardo Gutierrez. However, if you withdraw from the study, we will be unable to remove your responses to the survey once they have been submitted since we will have no way of identifying which responses are yours.

If you have questions about the research or wish to report a concern or complaint about the research, the Principal Investigator, Sanchi Malhotra MD may be reached at 213-209-1372. You may withdraw from this study at any time and discontinue participation without penalty. You are not waiving any legal claims, rights or remedies because of your participation in this research study. If you have questions regarding the rights of research subjects or if you have complaints or concerns about the research and cannot reach the Principal Investigator; or just want to talk to someone other than the Investigator, you may call the CHLA Human Subjects Protection Program at (323) 361-2265.
